# Kinetic and kinematic effects of asymmetric load carrying in the lower extremity

**DOI:** 10.1186/1757-1146-7-S1-A16

**Published:** 2014-04-08

**Authors:** Jasmien JE de Vette, Taeyong Lee, Xuezhen Song

**Affiliations:** 1 Department of Biomechanical Engineering, University of Twente, Enschede, 7500AE, The Netherlands; 2 Department of Biomedical Engineering, National University of Singapore, 119077, Singapore

## 

In everyday life, people have to transfer weight in all different forms from one place to the other. By the human body, this can be done in various ways. The impact in upper extremity of bearing load has been investigated throughout, especially with regard to load carried on the back or the front. However, the asymmetrical carrying of load has not been investigated in detail yet for the lower extremity. Hence, this investigation is established to study this topic in detail and to fill the gap of information regarding the asymmetrical bearing of weight.

Ten healthy individuals with no foot pathology (age: 20-30 years; 5 male, 5 female) were recruited, and the testing session were carried out at the Biomechanical Gait Analysis Laboratory at National University of Singapore. Subjects were asked to perform two sets of natural gait performance; first while carrying a hand-held bag, then by carrying a crossed sling-bag. The bag is weighted with 10% of the subjects’ body weight. Motion is recorded using an 8-camera VICON system and two force plates. Post processing of data is performed using Nexus 1.8.3 (Vicon, Oxford Metric, UK) and Polygon 3.5 (Vicon, Oxford Metric, UK).

An overall detailed view of the kinetic and kinematic effects of asymmetrical load carrying is given as a result of this research. Overall found weight is shifted towards the forefoot. At these points, ankle instability increases and therefore kinematic parameters in these areas are more tended to alter due to the extra weight. Overall, using a crossed sling bag is more favorable in terms of kinetic and kinematics in the lower extremity, especially showing in the heel-off and swing phase. Conclusions were the following. During the contact phases of the gait cycle, higher forces were found in the left side of the lower extremity in subjects bearing an extra weight on the right side of the body. Also, moments were found higher on the left side of the body, pointing out the counterbalancing effects to maintain posture during gait. All of these significant moments were pointed in medial direction. Ankle angles were found significantly asymmetric mostly in heel-off and pre-swing, where foot roll-off takes place.

